# Cardiovascular and cancer events in hyper-high-density lipoprotein cholesterolemic patients: a post hoc analysis of the MEGA study

**DOI:** 10.1186/1476-511X-13-133

**Published:** 2014-08-18

**Authors:** Haruo Nakamura, Kyoichi Mizuno, for the MEGA Study Group

**Affiliations:** Mitsukoshi Health and Welfare Foundation, 1-24-1, Nishi-shinjuku, Tokyo, 160-0023 Japan; Department of Medicine, Nippon Medical School, 1-5 Sendagi 1-chome, Bunkyo-ku, Tokyo, 113-8603 Japan

**Keywords:** Randomized prospective study, Hyper-high-density lipoprotein cholesterolemia, Statin, Coronary heart disease, Cancer

## Abstract

**Background:**

The prognosis for hyper-high-density lipoprotein (HDL) cholesterolemic patients has not been fully elucidated. We conducted a post hoc analysis of MEGA study data to investigate prospectively the incidence of cardiovascular events and cancer in hyper-HDL cholesterolemic patients.

**Methods:**

A total of 7832 patients with mild hypercholesterolemia were randomly allocated to either the National Cholesterol Education Program step 1 diet alone (*n* = 3966) or the diet plus pravastatin (*n* = 3866) and followed for 5 years. The incidences of coronary heart disease (CHD), CHD plus cerebral infarction (CI), cardiovascular disease (CVD), and cancer were calculated using the Cox proportional hazards model according to the level of HDL cholesterol (HDL-C).

**Results:**

CHD incidence was lower in patients with HDL-C >60–90 mg/dL (-52%, *p* = 0.0018) and HDL-C > 90 mg/dL (-46%, *p* = 0.4007) than in patients with HDL-C ≤ 60 mg/dL. The incidences of CHD, CHD plus CI, and CVD were significantly lower in patients with HDL-C >60–90 mg/dL than in those with HDL-C ≤ 60 mg/dL in both diet-alone and diet-plus-pravastatin groups. Cancer incidence was not increased in patients with HDL-C >60–90 mg/dL.

**Conclusion:**

Patients not receiving statin therapy should aim for a target HDL-C of between 60 and 90 mg/dL to achieve a significant reduction in CHD without the occurrence of adverse events.

**Trial registration:**

Clinical trials.gov NCT00211705.

## Background

A low level of high-density lipoprotein cholesterol (HDL-C) is one of the risk factors for cardiovascular disease (CVD) [1]. A number of research programs are underway with a view to increasing HDL-C and subsequently reducing atherosclerotic clinical events. However, the extent to which we should increase HDL-C and the prognosis for patients with hyper-HDL cholesterolemia has not been fully elucidated.

In Japan, 57% of individuals with HDL-C > 100 mg/dL have mutations of the cholesterol ester transfer protein (CETP) gene [2]. Furthermore, 37% of Japanese with HDL-C between 75 and 100 mg/dL have mutations of the CETP gene [3].

Some genetic mechanisms that increase HDL-C do not seem to lower the risk of myocardial infarction, for example a single nucleotide polymorphism (SNP) in the endothelial lipase gene [4]. In fact, lower plasma CETP activity was not associated with a reduced incidence of CVD in Framingham Heart Study participants [5]. Therefore it remains unclear whether a lower incidence of atherosclerotic disease is associated with increasing the HDL-C level or the genes that affect it.

In the primary prevention MEGA study [6] of the effects of low-dose pravastatin in Japanese patients with mild-to-moderate hypercholesterolemia and without CVD, total cholesterol and low-density lipoprotein cholesterol (LDL-C) were reduced by 12% and 18%, respectively. HDL-C was increased by 5.8% and, importantly, coronary heart disease (CHD) was significantly reduced by 33%.

The aim of the present study was to investigate prospectively the incidence of cardiovascular events and cancer in patients with hyper-HDL cholesterolemia in a post hoc analysis of the MEGA study and the results are reported in this article.

## Results

### Patient characteristics

The baseline characteristics of the patients according to HDL-C level are shown in Table 1. Of the 7832 patients, 2936 (37.5%) had HDL-C > 60 mg/dL and 239 (3.1%) had HDL > 90 mg/dL. The burden of atherosclerotic risk factors was less in patients with HDL-C > 60 mg/dL than in those with HDL-C ≤ 60 mg/dL.

**Table 1:** **Baseline characteristics**

	HDL-C (mg/dL)		
	≤ 60	> 60 to ≤ 90	> 90
*N*	4896	2697	239
Age (years)	58.1	58.7	58.2
Male (%)	37.8	21.4^*a*^	19.7^*a*^
Body mass index (kg/m^2^)	24.4	23.0^*a*^	21.4^*a*^
Hypertension (%)	44.8	37.3^*a*^	33.5^*a*^
Diabetes mellitus (%)	23.2	17.0^*a*^	15.5^*a*^
Total cholesterol (mg/dL)	242.3	243.1	242.5
LDL-C (mg/dL)	161.3	150.9^*a*^	126.4^*a*^
HDL-C (mg/dL)	48.4	70.3^*a*^	97.0^*a*^
Triglycerides (mg/dL)	172.2	110.2^*a*^	80.5^*a*^
Non–HDL-C (mg/dL)	193.9	172.8^*a*^	143.5^*a*^
Lipoprotein(a) (mg/dL)	23.9	26.1^*a*^	25.7^*a*^
Blood pressure (mmHg)			
Systolic	132.9	131.1^*a*^	130.0^*a*^
Diastolic	79.1	77.7^*a*^	77.8^*a*^
Fasting blood glucose (mg/dL)	110	105^*a*^	96^*a*^
Former or current smokers (%)	24.5	14.2^*a*^	13.4^*a*^

*HDL-C* high-density lipoprotein cholesterol, *LDL-C* low-density lipoprotein cholesterol.

^*a*^ *P* < 0.01 versus ≤ 60 mg/dL group.

Table 2 shows the incidence of major CVD and death in all patients according to HDL-C level.

**Table 2:** **Incidence of cardiovascular events according to high-density lipoprotein cholesterol level (5-year follow-up, all patients)**

	Baseline high-density lipoprotein cholesterol (mg/dL)	No. of cardiovascular events (cardiovascular events/1000 person-years)	Hazard ratio	95% CI	***p***	***p*** for trend
CHD	≤ 60	117/4896 (5.36)	1.0000		0.0066^*a*^	0.4007
	> 60 to ≤ 90	23/2697 (1.88)	0.4784	0.3011–0.7600	0.0018	
	> 90	2/239 (1.87)	0.5396	0.1280–2.2752	0.4007	
Stroke	≤ 60	72/4896 (3.29)	1.0000		0.8803^*a*^	0.9729
	> 60 to ≤ 90	27/2697 (2.21)	0.8870	0.5565–1.4140	0.6144	
	> 90	0/239 (0.00)	0.0000	0.0000–0.0000	0.9729	
Cerebral infarction	≤ 60	53/4896 (2.41)	1.0000		0.6707^*a*^	0.9768
	> 60 to ≤ 90	17/2697 (1.39)	0.7696	0.4333–1.3670	0.3717	
	> 90	0/239 (0.00)	0.0000	0.0000–0.0000	0.9768	
CHD plus cerebral infarction	≤ 60	166/4896 (7.65)	1.0000		0.0048^*a*^	0.1425
	> 60 to ≤ 90	40/2697 (3.28)	0.5733	0.4002–0.8212	0.0024	
	> 90	2/239 (1.87)	0.3453	0.0834–1.4299	0.1425	
Cardiovascular disease	≤ 60	199/4896 (9.21)	1.0000		0.0099^*a*^	0.1043
	> 60 to ≤ 90	54/2697 (4.44)	0.6496	0.4746–0.8891	0.0071	
	> 90	2/239 (1.87)	0.3094	0.0751–1.2742	0.1043	
Total deaths	≤ 60	82/4896 (3.66)	1.0000		0.0927^*a*^	0.2513
	> 60 to ≤ 90	26/2697 (2.09)	0.6335	0.4003–1.0023	0.0512	
	> 90	1/239 (0.91)	0.3091	0.0416–2.2980	0.2513	

*CHD* coronary heart disease, *CI* confidence interval.

^*a*^ *P* versus > 60 mg/dL group adjusted for age, sex, body mass index, hypertension, diabetes mellitus, low-density lipoprotein cholesterol, and smoking habit.

The risk of CHD, CHD plus cerebral infarction (CI), and all CVDs was significantly reduced in patients with HDL-C between 60 and 90 mg/dL, adjusted for age, sex, body mass index, hypertension, diabetes mellitus, LDL-C, and smoking habit. In contrast, the risk of stroke or CI was unchanged.

**Figure 1:**
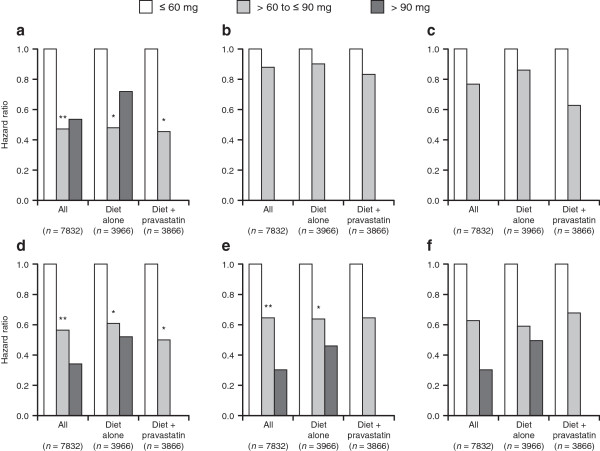
**Incidence of cardiovascular events according to high-density lipoprotein cholesterol level; in all patients (** ***n*** **= 7832), patients in the diet-alone group (** ***n*** **= 3966), and patients in the diet-plus-pravastatin group (** ***n*** **= 3866) (5-year follow-up): (a) coronary heart disease; (b) stroke; (c) cerebral infarction; (d), coronary heart disease plus cerebral infarction; (e) cardiovascular disease; and (f) total deaths.** *p < 0.05, **p < 0.01.

The incidence of cardiovascular events was also compared in the diet-alone and diet-plus-pravastatin groups separately (Figure 1). The hazard ratios for CHD, CHD plus CI, and CVD in the diet-alone and diet-plus-pravastatin groups were significantly lower in patients with HDL-C 60–90 mg/dL.

Conclusive evidence for a reduction in cardiovascular risk was not obtained for patients with HDL-C > 90 mg/dL. Two patients developed CHD during the follow-up period. One of them was a 68-year-old woman with normal blood pressure (106/72 mmHg), normal LDL-C (67.5 mg/dL) and triglycerides (81.7 mg/dL), no smoking habit, and HDL-C of 142.8 mg/dL. She had angina with coronary spasm, confirmed by coronary angiography. The other was a 59-year-old woman with high blood pressure (140–160/70–80 mmHg), normal LDL-C (130.6 mg/dL), and no smoking habit. Her HDL-C was 97.5 mg/dL. She was admitted to hospital and treated for angina confirmed by angiography.

**Figure 2:**
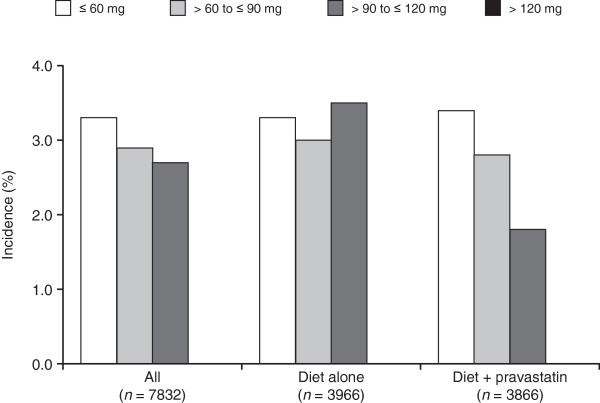
**Incidence of cancer according to high-density lipoprotein cholesterol level; in all patients (** ***n*** **= 7832), patients in the diet-alone group (** ***n*** **= 3966), and patients in the diet-plus-pravastatin group (** ***n*** **= 3866) (during follow-up).**

Table 3 shows the incidence of cancer during the average 5.3-year follow-up period according to HDL-C level. There were no significant differences in total incidence of cancer or in incidence of cancer at any site according to HDL-C level. Cancer incidence was further analyzed in the diet-alone group and diet-plus-pravastatin group separately. There were no significant differences in either group according to HDL-C level (Figure 2).

**Table 3:** **Incidence of cancer according to high-density cholesterol level (during follow-up, all patients)**

Cancer type	High-density lipoprotein cholesterol (mg/dL)	No. of patients (%)	***p***
All	≤ 60	164/4896 (3.3)	0.5654
	> 60 to ≤ 90	77/2697 (2.9)	
	> 90 to ≤ 120	6/225 (2.7)	
	> 120	0/14 (0.0)	
Digestive organs	≤ 60	80/4896 (1.6)	0.6998
	> 60 to ≤ 90	38/2697 (1.4)	
	> 90 to ≤ 120	5/225 (2.2)	
	> 120	0/14 (0.0)	
Respiratory organs	≤ 60	13/4896 (0.3)	0.7093
	> 60 to ≤ 90	10/2697 (0.4)	
	> 90 to ≤ 120	0/225 (0.0)	
	> 120	0/14 (0.0)	
Breast	≤ 60	14/3045 (0.5)	0.7375
	> 60 to ≤ 90	12/2119 (0.6)	
	> 90 to ≤ 120	0/185 (0.0)	
	> 120	0/7 (0.0)	
Female reproductive organs	≤ 60	18/3045 (0.6)	0.3139
	> 60 to ≤ 90	6/2119 (0.3)	
	> 90 to ≤ 120	0/185 (0.0)	
	> 120	0/7 (0.0)	
Other	≤ 60	46/4896 (0.9)	0.2190
	> 60 to ≤ 90	14/2697 (0.5)	
	> 90 to ≤ 120	1/225 (0.4)	
	> 120	0/14 (0.0)

## Discussion

The results of this prospective study show that the incidences of CHD, CHD plus CI, and CVD are reduced in patients with HDL-C 60–90 mg/dL (i.e. mild hyper-HDL cholesterolemia). This finding is consistent with the results of a previous prospective study on Japanese-American men in Hawaii [7] and an observational prevalence study on the population of Western Japan [8]. Elderly Japanese-American men in Hawaii with heterozygous CETP deficiency and intermediate HDL-C (41–60 mg/dL) have an increased prevalence of CHD [9]. However, the present study shows that patients with increased HDL-C (>60 mg/dL) have a low risk of CHD regardless of the presence of CETP abnormality.

The incidence of stroke and total death in this study did not differ significantly between patients with HDL-C ≤ 60 mg/dL and those with HDL-C > 60 mg/dL.

A recent study showed that some genetic mechanisms that increase HDL-C do not seem uniformly to lower the risk of myocardial infarction [4]. The authors tested for an SNP in endothelial lipase. In most patients, the presence of this SNP did not increase HDL-C to > 60 mg/dL. Neither the mass nor activity of CETP was available in this study. Plasma CETP activity was measured in 1,978 participants of the Framingham Heart Study, and lower CETP activity was found to be associated with greater risk of CVD [5]. However, there seems to be no association between CETP activity and HDL-C level, which remained to be at < 60 mg/dL.

Further investigations of the association between three CETP genotypes and the incidence of CHD have shown higher HDL-C to have a weakly inverse association with coronary risk [10]. Also, CETP-deficient families, including heterozygous persons, have increased levels of HDL-C (>60 mg/dL) and no evidence of premature atherosclerosis [11].

A higher HDL-C level (>60 mg/dL) seems to be the threshold for preventing CHD. However, two patients in the diet-alone group developed CHD despite having HDL-C > 90 mg/dL. One of these patients had hypertension and the other had HDL-C > 120 mg/dL, and both developed spastic angina confirmed by coronary angiography, which was relieved by the coadministration of nitrate and statin.

High-density lipoprotein plays several roles, including reverse cholesterol transport and endothelial, antioxidative, and immunological functions. CETP inhibitors increase HDL by promoting reverse cholesterol transport [12], with small HDL remaining [13]. Therefore patients with spastic angina may have had endothelial dysfunction. No cases of angina were found in the diet-plus-pravastatin group in the present analysis. Therefore the precise mechanism should be clarified under further experimental conditions.

These findings suggest that the target threshold HDL-C for patients not receiving statin therapy should be between 60 and 90 mg/dL for them to achieve a significant reduction in CHD without the occurrence of adverse events.

Regarding the incidence of cancer, there were no significant differences in the incidence of cancer at any site according to HDL-C level. Cancer-related mortality was reduced with statin use in previous studies [14, 15].

To exclude selection bias, we separated the diet-alone group from the diet-plus-pravastatin group. There were no significant differences in the total incidence of cancer or the incidence of cancer at different sites according to HDL-C level.

The limitations of this study include the relatively short follow-up period and the small number of participants. However, the study included participants from all over Japan (from Hokkaido in the north to Okinawa in the south) approximately in proportion to the population in different areas.

Three percent of patients with mild hypercholesterolemia (total cholesterol ≥ 220 mg/dL) had hyper-HDL-C cholesterolemia (HDL-C > 90 mg/dL) and 38% had mildly increased HDL-C (>60 mg/dL). These figures are considered to be representative for Japanese. Furthermore, figures for the incidence of cardiovascular events and cancer were reliable because of the high follow-up rate (99.4%) [6].

Another limitation to this study is the lack of information on the markers or mechanisms for the increase in HDL-C. However, this was not a purpose of the original investigation. Also, a previous study has confirmed the reduction in cardiovascular events in patients with high HDL-C irrespective of the presence or absence of CETP deficiency [8].

## Conclusions

Patients with high HDL-C (>60 mg/dL) have a low incidence of cardiovascular events, and the incidence of cancer was not increased at any site.

## Methods

### Patients

All 7832 MEGA study patients were included in this post hoc analysis. The details of the MEGA study have been reported [6]. Briefly, this prospective randomized, open-label, blinded endpoint study was conducted in Japan between 1994 and 2004 in men and postmenopausal women aged 40–70 years with a moderate level of hypercholesterolemia (total cholesterol, 220–270 mg/dL) and without a history of CHD or cerebrovascular disease. A total of 3966 patients were randomly assigned to the diet-alone group and 3866 patients to the diet-plus-pravastatin group. Patients in both groups followed the National Cholesterol Education Program step 1 diet. Physicians and dieticians estimated that > 70% of patients adhered to the diet, with no significant differences between the two groups. The dose of pravastatin was 10–20 mg/day, the approved dose range in Japan.

The primary endpoint was incidence of CHD defined as a composite of fatal and non-fatal myocardial infarction, angina, cardiac sudden death, and revascularization procedure. Secondary endpoints were stroke, CHD plus CI, all cardiovascular events, and total mortality.

Patients were examined at 1, 3, and 6 months after the start of follow-up, and every 6 months thereafter. All patients provided written informed consent. The trial was conducted according to the ethical principles of the Declaration of Helsinki and the Japanese Ministry of Health, Labour and Welfare ordinance regarding post-marketing surveillance. The MEGA study is registered at clinical trials.gov as trial no. http://NCT00211705.

### Biochemical measurements

Blood samples were taken in the fasting state before randomization and at the start of the study; 1, 3, and 6 months after the start of follow-up; and every 6 months thereafter. Total cholesterol and triglycerides were measured by enzymatic methods. LDL-C was calculated by using Friedewald’s formula [16]. All lipid values were measured centrally in a blinded manner at Special Reference Laboratory (SRL, Hachioji, Tokyo, Japan), which is certified for major lipid measurement by the Centers for Disease Control (Atlanta, GA, USA). Biological markers related to the change in HDL-C were not analyzed.

The subgroups of this study were classified as follows. In the previous analysis, no significant differences were found in the incidence of CHD between patients with HDL-C < 55 mg/dL and patients with HDL-C ≥ 55 mg/dL, using the Cox proportional hazards model [6]. Therefore the cut-off level for hyper-HDL cholesterolemia was increased to 60 mg/dL, with mild hyper-HDL cholesterolemia and hyper-HDL cholesterolemia arbitrarily considered as HDL-C > 60 and > 90 mg/dL, respectively.

### Statistical analyses

Baseline characteristics were compared across three groups (patients with HDL-C ≤ 60 mg/dL, 60 < HDL-C ≤ 90 mg/dL, and HDL-C > 90 mg/dL) using the chi-squared test for categorical variables or the Wilcoxon rank sum test for continuous variables. The incidence of cardiovascular events and cancer was compared in all patients, patients in the diet-alone group, and patients in the diet-plus-pravastatin group. Hazard ratios and their 95% confidence intervals were calculated by using the Cox proportional hazards model adjusted for baseline risk factors, which were age, sex, body mass index, hypertension, diabetes, LDL-C, and lipoprotein(a). All *p* values were two-sided and a significance cut-off of 0.05 was used.

## Abbreviations

CETP: Cholesterol ester transfer protein
CHD: Coronary heart disease
CI: Cerebral infarction
CVD: Cardiovascular disease
HDL-C: High-density lipoprotein cholesterol
LDL-C: Low-density lipoprotein cholesterol
SNP: Single nucleotide polymorphism.
